# Reinvigorating the Role of Science in Democracy

**DOI:** 10.1371/journal.pbio.1001553

**Published:** 2013-05-07

**Authors:** Andrew A. Rosenberg, Michael Halpern, Seth Shulman, Celia Wexler, Pallavi Phartiyal

**Affiliations:** Center for Science and Democracy, Union of Concerned Scientists, Cambridge, Massachusetts, United States of America

## Abstract

Private and political interests routinely conspire to sideline and misrepresent science and evidence in the public policy process. The Center for Science and Democracy, a new initiative at the Union of Concerned Scientists, endeavors to change this dynamic to strengthen the role of science in decision making.

## Introduction

Good policy decisions require reasonable and robust debate grounded in the best possible information. And yet, it is no secret that political discourse around the world, with the United States as a prime example, has become increasingly, and even bitterly, partisan. Many governments with parliamentary systems are formed by fragile coalitions, and political discourse is heated. In the United States, spending on lobbying and campaigns is at an all-time high [1], giving special interests immense access to lawmakers. The public approval rating of the U.S. Congress stands at an all-time low [2], but disapproval of government is by no means confined to the American electorate [3].

In too many cases, science and scientific advice have been marginalized in public policy debates around the world, ranging from natural resource use (e.g., fisheries, forestry) to environmental impacts (e.g., climate, air, water, mining, or transportation) to public health and safety (e.g., pharmaceuticals, tobacco use, food and product safety).

In the United States, where the new Center for Science and Democracy (www.ucsusa.org/center-for-science-and-democracy/) at the Union of Concerned Scientists will be focusing our efforts, misinformation on scientific issues abounds, from local city councils to the halls of the U.S. Congress, fueled by a never-ending news cycle in which anyone with an internet connection can pose as an expert. Instead of seeking the best available science, elected officials seek analyses that support the policies they wish to put forward. To make matters worse, even scientific and technical facts that are accepted as established knowledge in virtually every other developed nation are commonly dismissed in American discourse, on topics as diverse as climate change and the safety of vaccines. When decision makers cannot agree on even the basic facts underlying a problem and the science itself is politicized, good policy outcomes become significantly less likely.

Regrettably, these developments come at a time when the public and decision makers face some of the most complex and daunting problems in our history: mitigating and adapting to the impacts of global warming, finding sustainable ways to feed, power, and transport ourselves, and reducing the threat of catastrophic war. Against this backdrop, the U.S. government's inability to implement sound policies on even the most straightforward science-based issues such as climate change is particularly troubling. When we allow policy makers to treat scientific advice, based upon a well-developed and transparent scientific process, as just another special-interest opinion, we jeopardize not only the opportunity to make good policy decisions, but also our health, environment, and quality of life.

We possess the intellectual capacity and infrastructure to restore science to its rightful place in democratic decision making. So, what steps do we need to take toward this end?

## Science and Democracy: A Powerful Partnership

**Figure 1:**
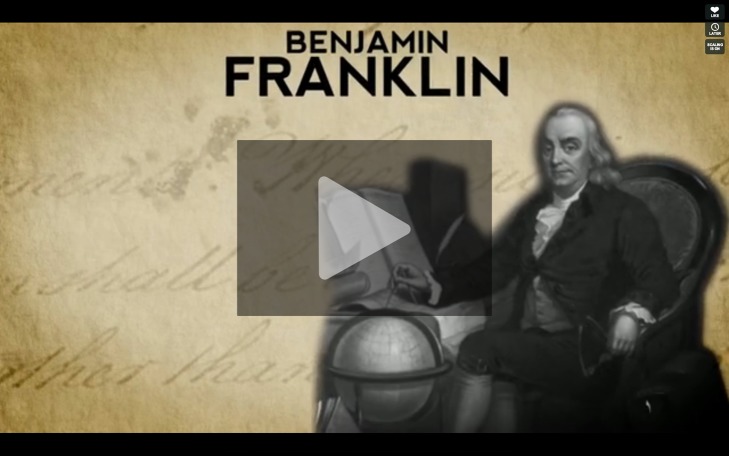
Several of the American Founding Fathers were citizen scientists who invoked scientific methods and principles in developing our system of governance. Video source: http://www.ucsusa.org/center-for-science-and-democracy/why-a-center-for-science-and-democracy.html.

Science and democracy, when working together, have proven to be one of the great partnerships over the past 200 years as democratic governments have developed around the world, producing enormous rewards in public health and welfare. Indeed, the American Founding Fathers in the eighteenth century, influenced by the ideas of the Enlightenment, used scientific principles to shape our system of government (see video at http://www.ucsusa.org/center-for-science-and-democracy/why-a-center-for-science-and-democracy.html). Many are aware of Benjamin Franklin's experiments on electricity, but Franklin was not alone. Thomas Jefferson called “the tranquil pursuit of science” his “supreme delight.” He collected and classified fossils, was a prolific inventor, and a student of mathematics, science, agriculture, and architecture [4]. John Adams spoke of the “science of government.” In a debate with Benjamin Franklin in 1776, Adams invoked the principle of mechanical equilibrium to argue for his conception of our government's system of checks and balances (Figure 1)—designed, at least in part, to ensure policies based on verified, trustworthy evidence [4].

Concepts such as transparency, a rigorous examination of ideas, review and critique by technically qualified peers, free speech and open exchange, and protection against retaliation for one's beliefs (or findings) are central to the health of both science and democratic government. Not surprisingly then, when these concepts are not respected, from restricting the ability of teachers to present scientific evidence on evolution to unethical medical trials on at-risk populations, both science and democracy suffer.

The record shows the progress we can make when science plays a key role in the policy-making process. In 2002, for example, scientists and public health services in the United States and other countries quickly contained the outbreak of severe acute respiratory syndrome, better known as SARS, protecting the public and minimizing fatalities [5],[6]. Time and again, government investments in medical research and vaccination programs based on data and careful analysis have led to the successful containment of diseases such as smallpox, polio, cholera, diphtheria, and rubella that killed and crippled our grandparents and their ancestors. Science-based laws, such as the U.S. Clean Air Act and Clean Water Act, and their counterparts around the world (such as the EU Water Directive or the Canada Environmental Protection Act) have effectively reduced deadly pollutants and saved hundreds of thousands of lives over the past four decades [7],[8].

Or consider the important role science played in developing policy to combat the hole in the planet's ozone layer. In the 1970s, Drs. Sherry Rowland and Mario Molina discovered that chlorofluorocarbons (CFCs), chemicals widely used in deodorants, refrigerants, and hairsprays, were destroying the stratospheric ozone layer that protects us from ultraviolet rays. Initially, DuPont Corporation and other CFC manufacturers reacted by attempting to malign the scientists' professional reputations, a time-honored practice at least since Rachel Carson first wrote about DDT in the 1960s [9]. One article in the trade publication *Aerosol Age* even went so far as to accuse the scientists of being K.G.B. agents out to destroy capitalism [10]. For a while, the attacks worked. Dr. Rowland recalled that he was not invited to speak to a single university chemistry department about his work for the next decade [10]. But in 1985, British Antarctic Survey researchers Joseph Farman, Brian Gardiner, and Jonathan Shanklin documented the existence of a hole in the ozone layer over the Antarctic [11], providing incontrovertible evidence of Rowland and Molina's claims about CFCs' damaging effects. Notably, Rowland, Molina, Farman, Gardiner, Shanklin, and other scientists provided the scientific evidence that laid the groundwork for action. As public concern mounted, unilateral action by several countries eventually led to an international agreement in 1987 to address the problem by committing to a phase out of ozone-depleting compounds—a response that has since put the ozone hole on the path to recovery [12].

## Strengthening Science and Democracy

It's important to recognize that even the strongest laws, like the U.S. Clean Water Act, face constant political pressure from interest groups who see governmental regulations as a threat to their bottom line. In the United States, Congress tends to respond to this pressure by restricting agency budgets and constraining agency action. For example, while the Clean Water Act and the Safe Drinking Water Act would seem to pertain to activities such as hydraulic fracturing (fracking) of shale for oil and gas because of the chemicals used and the wastewater generated from these practices, Congress exempted fracking operations from regulation when it passed the Energy Policy Act of 2005. This essentially delegated regulation of fracking practices and disclosure of chemicals to individual U.S. states. In fact, fracking operations are exempt from other federal statutes such as provisions of the Clean Air Act as well, effectively removing much of the basis for federal oversight. Recently, Common Cause, a U.S. public interest group, reported that a faction of the natural gas industry had spent more than $747 million dollars over the last decade to persuade federal authorities that fracking poses little risk and should be left to states to regulate as they see fit [13].

This is not an isolated example. Industry influence or political pressure has resulted in public-policy decisions on a wide range of issues, from controlling soot from smokestacks and exhaust pipes to the availability of contraception methods like the morning-after pill. In each of these cases, science has been pushed aside in the name of political expediency.

To restore public confidence in, and support for, the use of independent science in public policy making, the Union of Concerned Scientists established the Center for Science and Democracy. We recognize that this is a formidable task with outcomes that will be realized over the long term. Success will require a multipronged approach too, outlined below, which we hope others will join us in.

*Empower scientists to engage more deeply with their communities.* The Center for Science and Democracy is building upon the 20,000+ network of scientists, engineers, and health professionals the Union of Concerned Scientists has cultivated over the past two decades. We are equipping scientists with the skills they need to effectively communicate their expertise to decision makers, media, and the public through targeted training sessions, often in coordination with major scientific gatherings. Furthermore, we are connecting trained scientists with opportunities to help local and national decision makers use science more effectively in crafting policy. We are reaching out to science graduate students, young scientists, and innovators in order to create the next generation of citizen scientists.

*Provide citizens with better access to reliable scientific information.* Citizens and journalists need better tools to distinguish evidence-based information from propaganda. We are exploring ways to better deliver reliable and accessible scientific information, and expertise, to those who need it.

*Create opportunities for citizens, educators, scientists, and decision makers to engage on science-based policy issues.* We are convening a Science and Democracy Forum series that will bring together experts from across disciplines and stakeholders to develop nonpartisan solutions to complex, science-based challenges. In partnership with universities and other institutions, we will convene a series of seven to nine forums over the next three years, tackling a variety of issues (for example, decision making on fracking, available at http://www.ucsusa.org/center-for-science-and-democracy/events/community-decisions-on-fracking.html).

*Cultivate a network of science and democracy opinion leaders to speak out about the benefits of science and evidence-based policies in our democracy.* Ministers, corporate executives, military leaders, science educators, and elected officials from diverse political perspectives can offer powerful testaments to the benefits of science-based decision making in their communities. Political leaders are especially important in spreading this message to rebut the flawed idea that science is partisan.

**Figure 2:**
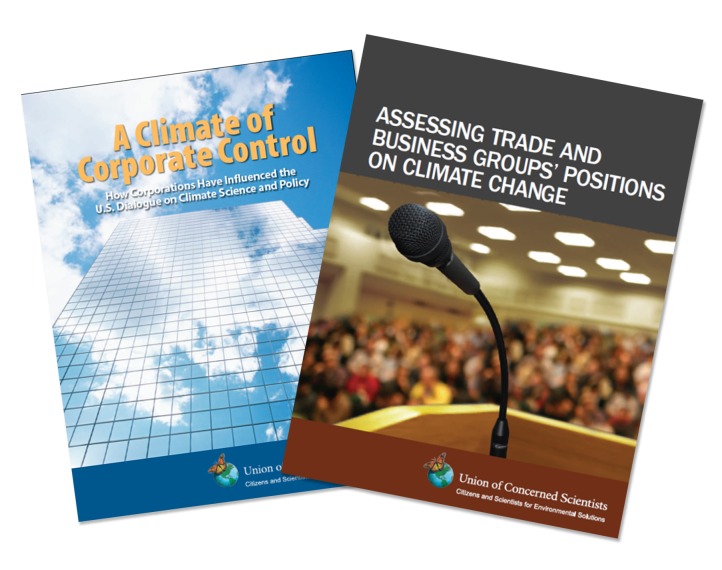
The Center for Science and Democracy at the Union of Concerned Scientists strives to hold corporations accountable by exposing their influence on science-based public policy.

*Increase government and corporate accountability by exposing and publicizing instances in which science is misrepresented or misused.* We will assess the use and misuse of scientific information in public discourse on policy-relevant issues, exploring, for example, how corporations influence the national dialogue about key science-policy issues, or the current and past states of science discourse in Congress (Figure 2; [14],[15]).

Democratic societies around the world need the persistent and energetic engagement of scientists and nonscientists alike. While our focus is the United States, the approaches detailed here are broadly applicable to the international science community, working with citizens in democracies around the world. Of course we must ourselves remain open to exchange of ideas, learning, innovating, and trying new approaches as we encourage and enable more politicians and opinion leaders to trust in and rely upon facts, no matter what divergent political viewpoints they may hold.

The stakes are high. As Carl Sagan put it: “Whether we will acquire the understanding and wisdom necessary to come to grips with the scientific revelations of the twentieth century will be the most profound challenge of the twenty-first century” [16].

## Abbreviations

CFCs: chlorofluorocarbons
SARS: severe acute respiratory syndrome
